# Adjuvants for Coronavirus Vaccines

**DOI:** 10.3389/fimmu.2020.589833

**Published:** 2020-11-06

**Authors:** Zhihui Liang, Haoru Zhu, Xin Wang, Bo Jing, Zifan Li, Xinyu Xia, Hongwu Sun, Yun Yang, Weiting Zhang, Li Shi, Hao Zeng, Bingbing Sun

**Affiliations:** ^1^ State Key Laboratory of Fine Chemicals, Dalian University of Technology, Dalian, China; ^2^ School of Chemical Engineering, Dalian University of Technology, Dalian, China; ^3^ National Engineering Research Center of Immunological Products, Department of Microbiology and Biochemical Pharmacy, College of Pharmacy and Laboratory Medicine, Third Military Medical University, Chongqing, China; ^4^ NCPC Genetech Biotechnology Co., Ltd., Shijiazhuang, China; ^5^ Basic Research Department, Shanghai Zerun Biotechnology Co., Ltd., Shanghai, China

**Keywords:** coronavirus disease 2019, SARS-CoV-2, adjuvant, coronavirus vaccine, aluminum salt

## Abstract

Vaccine development utilizing various platforms is one of the strategies that has been proposed to address the coronavirus disease 2019 (COVID-19) pandemic. Adjuvants are critical components of both subunit and certain inactivated vaccines because they induce specific immune responses that are more robust and long-lasting. A review of the history of coronavirus vaccine development demonstrates that only a few adjuvants, including aluminum salts, emulsions, and TLR agonists, have been formulated for the severe acute respiratory syndrome-associated coronavirus (SARS-CoV), Middle East respiratory syndrome-related coronavirus (MERS-CoV), and currently the SARS-CoV-2 vaccines in experimental and pre-clinical studies. However, there is still a lack of evidence regarding the effects of the adjuvants tested in coronavirus vaccines. This paper presents an overview of adjuvants that have been formulated in reported coronavirus vaccine studies, which should assist with the design and selection of adjuvants with optimal efficacy and safety profiles for COVID-19 vaccines.

## Introduction

Coronaviruses (CoVs) are single-stranded RNA viruses characterized by club-like spikes that can potentially cause severe respiratory disease in humans (1, 2). The outbreak of severe acute respiratory syndrome (SARS) caused by the SARS-CoV resulted in more than 8000 confirmed infections, with an overall case fatality rate of 10% in 2002 (3). The Middle East respiratory syndrome (MERS)-CoV continues to cause deaths with increasing geographical distribution and a 34.4% case fatality rate, according to the World Health Organization (WHO) (4). Most recently, the coronavirus disease 2019 (COVID-19) caused by SARS-CoV-2 has spread globally, with over 33 million confirmed cases as of October 2020 (5). Considering the challenges to global health systems and the far-reaching consequences on the world economy, there is an urgent need to develop effective and safe vaccines that can be quickly deployed on a global scale (2, 6).

Vaccine candidates are currently under development using different platforms, such as inactivated vaccines, recombinant protein vaccines, live-attenuated vaccines, viral vector (adenovirus) vaccines, DNA vaccines, and mRNA vaccines (2, 6, 7). Adenovirus-vector could induce potent immunological responses due to the presence of viral proteins and stimulation of innate immunity sensors, *e.g.*, toll-like receptors (8). Nucleic-acid vaccines, *e.g.*, DNA and mRNA vaccines, encode the virus’s spike protein, intrinsically could engage innate immunity that instructs induction of immune protection (9). However, these platforms haven’t been used in licensed human vaccines before. In other platforms, subunit or inactivated antigens were used, but these antigens lack the immunological profiles that mediate the enhanced adaptive immunity. Thus, in these CoV vaccines, they require the addition of adjuvants for directing the types and magnitude of immune responses (10). In previously reported exploratory and pre-clinical CoV vaccine studies, adjuvants such as aluminum salts, emulsions, and toll-like receptor (TLR) agonists, have been used in vaccine formulations for studies with various animal models (**Table 1**). The adjuvants AS03, MF59, and CpG 1018 have already been used in licensed vaccines (28) and have been committed by GlaxoSmithKline, Seqirus, and Dynavax to be available for COVID-19 vaccine development (29). When combined with subunit and specific inactivated antigens (30, 31), adjuvants with various characteristics elicit distinctive immunological profiles with regard to the direction, duration, and strength of immune responses. Thus far, there are at least 40 candidate vaccines in clinical trials and 149 vaccines in preclinical evaluation, of which 67 subunit and 15 inactivated COVID-19 vaccines have being developed (32). Among these adjuvants, alum have been formulated with S protein or RBD to induce neutralizing antibody production (17, 18), which has suggested to be associated with protection against SARS-CoV-2 (15, 16, 24). However, alum lacks the capability to promote the activation of CD4^+^ and CD8^+^ T cell responses, which has been demonstrated to coordinate with the antibody responses to provide protective immunity against the SARS-CoV-2 (33). Other adjuvants, *e.g.*, emulsion adjuvants and TLR agonists, which have been shown to induce both humoral and cellular immune responses could be more favorable. However, no phase III clinical trial results of COVID-19 vaccines are published so far, thus, there is no direct evidence to indicate which type of immune response induced by vaccine plays a more critical protective role in SARS-CoV-2 infection. Knowing these uncertainties, an overview of previous CoV vaccine studies using different adjuvants would be indispensable for the design and development of a COVID-19 vaccine.

**Table 1:** Alum based adjuvants used in the coronavirus vaccine formulations under exploratory and pre-clinical investigations.

Adjuvant Type	Platform	Adjuvant	Antigen	Antigen Dose	Immunological response								Route	Immunization Schedule	Safety	Animal model	Ref.
					Nab	IgG_1_	IgG_2a_	IgA	Th1 cytokines	Th2 cytokines	Th17 Cytokines	CD8^+^ T cell response					
Alum based adjuvants	Inactivated vaccines	Aluminum hydroxide	Doubly inactivated (formalin and UV) whole SARS virus	0.125/0.25/0.5/1 μg	√	N/A	N/A	N/A	N/A	N/A	N/A	N/A	i.m.	2	Pulmonary immunopathology	BALB/c or C57BL/6 mice	(11)
	Inactivated vaccines	Aluminum hydroxide	Purified beta propiolactone inactivated whole SARS virus	2 μg	√	N/A	N/A	N/A	N/A	N/A	N/A	N/A	i.m.	2	N/A	BALB/c or C57BL/6 mice	(11)
	Inactivated vaccines	Aluminum hydroxide gel	UV-inactivated purified SARS-CoV	10 μg	√	√	×	×	√	√	N/A	N/A	s.c.	2	N/A	BALB/c mice	(12)
	Inactivated vaccines	Aluminum hydroxide	SARS-CoV (formaldehyde and U.V. inactivation)	0.3~1 μg	√	N/A	N/A	N/A	√	√	N/A	N/A	s.c.	2	N/A	CD1 mice	(13)
	Inactivated vaccines	Alum	Double-inactivated SARS-CoV (DIV)	0.2 μg	√	√	×	N/A	×	√	N/A	N/A	Footpad injection	2	Lung immunopathology	BALB/c AnNHsd mice	(14)
	Inactivated vaccines	Aluminum hydroxide	SARS-CoV-2	Mice/Rats:1.5/3/6 μg; Monkeys:1.5/6 μg	√	N/A	N/A	N/A	×	×	N/A	N/A	i.m./i.p.	Mice/Rats:2; Monkeys:3	×	BALB/c mice; Wistar rats; Rhesus macaques	(15)
	Inactivated vaccines	Aluminum hydroxide	SARS-CoV-2	2/4/8 (μg/dose)	√	N/A	N/A	N/A	N/A	N/A	N/A	N/A	i.p./i.m	1/2/3	No acute toxicity/No Systemic anaphylax/No long-term toxicity	Rats; Mice guinea pigs; Rabbits, Cynomol-gus monkeys; Rhesus macaques	(16)
	Subunit vaccines	Alhydrogel	SARS S protein	3 μg	√	N/A	N/A	N/A	N/A	N/A	N/A	N/A	i.m.	2	N/A	BALB/c mice	(17)
	Subunit vaccines	Alhydrogel	MERS S protein	1/3/10 μg	√	N/A	N/A	N/A	N/A	N/A	N/A	N/A	i.m.	2	N/A	BALB/c mice	(17)
	Subunit vaccines	Aluminum hydroxide	MERS-CoV S rRBD Protein	10 μg	√	√	√	N/A	×	√	N/A	N/A	i.m.	3	N/A	BALB/c mice	(18)
	Subunit vaccines	Alhydrogel	SARS-CoV S318–510 fragment	8 μg	√	√	×	N/A	×	N/A	N/A	N/A	s.c.	2	N/A	129S6/SvEv mice	(19)
	Subunit vaccines	Alhydrogel	Ectodomain of SARS-CoV S glycoprotein (ΔTMS)	1//5 μg	√	N/A	N/A	N/A	N/A	N/A	N/A	N/A	i.m.	1	N/A	CD1 mice	(20)
	Subunit vaccines	Aluminum hydroxide	S glycoprotein of SARS-CoV	0.25/0.5/1/2 μg	√	N/A	N/A	N/A	N/A	N/A	N/A	N/A	i.m.	2	N/A	BALB/c or C57BL/6 mice	(11)
	Subunit vaccines	Aluminum hydroxide	MERS-CoV rRBD protein S367-606	100/50 μg (primed)+50/25 μg (boosted)	√	N/A	N/A	N/A	N/A	N/A	N/A	N/A	i.m.	3	N/A	Monkeys	(21)
	Subunit vaccines	Aluminum phosphate	MERS-CoV S1 protein	10 μg	√	N/A	N/A	N/A	N/A	N/A	N/A	N/A	i.m.	2	N/A	BALB/c mice	(22)
	Subunit vaccines	Imject™ Alum	MERS-CoV RBD-dimer	10 μg	√	N/A	N/A	N/A	×	×	N/A	N/A	i.m.	3	N/A	BALB/c mice	(23)
	Subunit vaccines	Aluminum hydroxide gel	RBD of SARS-CoV-2 spike protein	0.1-20 μg	√	N/A	N/A	N/A	N/A	N/A	N/A	N/A	i.m	1/2/3	Safe	BALB/c and C57BL/6mice; Rabbits;Non-human primates (Macaca mulatta)	(24)
	VLP vaccines	Aluminum hydroxide	SARS-CoV S glycoprotein and E, M, and N proteins	2 μg	×	N/A	N/A	N/A	N/A	N/A	N/A	N/A	i.m.	2	N/A	BALB/c or C57BL/6 mice	(11)

Annotation:

**S protein** is the spike glycoprotein of coronavirus and a main target for neutralizing antibodies (25);

**RBD** is the receptor-binding domain (RBD) of S protein, which could directly interact with the angiotensin-converting enzyme 2 (ACE2) receptor on host cells (25);

**N protein** is the highly conserved nucleocapsid protein of coronavirus and regulates RNA replication and transcription (26);

**M protein** (membrane protein) is the most abundant protein on the coronavirus surface and mediates virus assembly (25);

**E protein** (envelope protein) is an integral membrane protein and plays a pivotal role in virus envelope formation (27).

**Nab** represents the neutralizing antibodies, and N/A means not available.

The SARS-CoV-2 is a novel strain of the coronavirus, and very little is known about its epidemiology and pathogenesis. Therefore, extreme cautions should be taken when considering vaccine formulations that can achieve the desired efficacy and safety profiles. The selection of adjuvants should consider the magnitude, affinity, isotype, and durability of antibodies that are critical for coronavirus vaccine developments (34). It should be noted that low antibody production may lead to antibody-dependent enhancement (ADE) manifested by severe liver damage and enhanced infection (35), while high affinity neutralizing antibodies could help to avoid ADE. Additionally, the proper application of adjuvants also depends on the choice of antigens. The full-length S protein is more likely to trigger ADE due to mild antibody production (36). In comparison, the N protein is generally highly conserved, and it is associated with the ability to induce cytotoxic T lymphocytes (CTL). However, N protein could potentiate pro-inflammatory cytokine production and lead to severe lung pathology (37). In addition, previous study on respiratory syncytial virus (RSV) vaccine also indicated that immunization with whole inactivated virus could lead to vaccine-associated enhanced respiratory disease (VAERD), manifested by allergic inflammation and Th2 type immune responses (38). Altogether, these studies suggest that vaccines formulated with various antigen isotypes may require proper adjuvant selection to achieve the desired immune protection. In this paper, we reviewed adjuvants that have already been incorporated in the coronavirus vaccines under exploratory and pre-clinical investigations. By reviewing the vaccine formulations and the types of immune responses that were induced, we provide information that will enable proper adjuvant selection for COVID-19 vaccines to facilitate rapid vaccine delivery.

## Aluminum Salt-Based Adjuvants

Aluminum salt-based adjuvants (alum) were the first adjuvants used in licensed human vaccines. They are still the most widely used because of their wide-spectrum ability to strengthen immune responses and their excellent track record of safety (39–41). In limited coronavirus vaccine studies, it has been suggested that neutralizing antibody against the spike protein might be mechanistically correlated with immune protection (42). When alum was formulated with S protein or receptor-binding domain (RBD), it significantly enhanced humoral immune responses. This was demonstrated by higher titers of serum IgG_1_, increased high affinity viral neutralizing antibodies, and the generation of long-lasting memory B cells in mice (13, 17–19). Additionally, Alum was formulated with the inactivated and VLP vaccines containing E, M, and N proteins (11, 12, 14) (**Table 1**) that showed enhanced IgG_1_ and neutralizing antibody titers (14) and prolonged durability (12). Studies also demonstrated that alum adjuvant plays an essential role in the dose-sparing of CoV vaccines. In a SARS S protein subunit vaccine, the alum-adjuvanted S protein (1 μg) group showed neutralizing antibody titers similar to or higher than the non-adjuvanted S protein (50 μg) group. The alum-adjuvanted S protein (5 μg) group showed a geometric mean titer (GMT) twice as high as the non-adjuvanted S protein (50 μg) group (20). It should also be noted that different types of alum were selected in the studies, including Alhydrogel, which is chemically crystalline aluminum oxyhydroxide (43), aluminum hydroxide (11), aluminum phosphate (22), and Imject™ Alum (23), which is a mixture of aluminum hydroxide and magnesium hydroxide. Even though there is no specific description regarding the aluminum hydroxide in reported literature (11, 18, 21), it can also be referred to Aluminum oxyhydroxide (44). However, these studies lacked systematic comparisons with regards to their adjuvanticity and how various alum-based adjuvants differed in their ability to induce neutralizing antibodies.

It is worth noting that inactivated SARS-CoV or S protein-based vaccines are associated with Th2-type immunopathology, which is characterized by an increase in eosinophils and inflammatory infiltrates (14, 30, 37, 45). Moreover, the addition of alum adjuvant exacerbated the immunopathologic reactions (14, 45). In alum-adjuvanted SARS-CoV double-inactivated vaccine (DIV), there was a skew in the N or S protein-specific antibodies toward IgG_1_, when compared with the more balanced antibody production in the nonadjuvanted DIV vaccine (14). These observations raise significant concerns regarding the safety of adjuvanted coronavirus vaccines. On the other hand, it has been shown that alum can reduce immunopathology in SARS-CoV vaccines containing either a double-inactivated virus or S protein (11). Furthermore, in a recent study, a purified inactivated SARS-CoV-2 vaccine (PiCoVacc) adjuvanted with aluminum hydroxide conferred complete protection in non-human primates (rhesus macaques) with potent humoral responses but without lung immunopathology (15). This finding raises the question of the mechanism of eosinophilic immunopathology. While commonly thought of as the product of Th2 responses, recent studies have indicated that tissue eosinophilia can also be controlled by Th17 responses (46). Thus, the proper selection of CoV antigens and adjuvants that can shift host responses away from a Th17-bias appears to be critical. In addition, other studies have demonstrated that the Th2 immunopathology may be associated with SARS N or S protein that results in enhanced eosinophilic immunopathology (11, 37, 47). However, more studies are required, as the preliminary data is limited. Additionally, the Th-2–biased immune responses may raise the concern on vaccine-enhanced respiratory disease (VAERD) (38, 48), however, there are no evidences that alum-adjuvanted CoV vaccines show the effect.

When alum was used as an adjuvant in CoV vaccines (**Table 1**), there was a lack of Th1 CD4^+^ T cell and cytotoxic CD8^+^ T cell immune responses, which is typical for alum-adjuvanted vaccines (49). However, recent study has demonstrated that the SARS-CoV-2–specific adaptive immune response correlated with milder disease, indicating that coordinated CD4^+^ and CD8^+^ T cell responses play a synergistic effect in the protective immunity of COVID-19 (33). Several other adjuvants, which are capable of inducing more balanced Th1/Th2 or Th1-biased immune responses, have been formulated in CoV vaccines and will be discussed in the following sections.

## Emulsion Adjuvants

The emulsion adjuvants, MF59, and AS03 have already been used in licensed human vaccines to improve the immunogenicity of the antigens (50, 51). Compared with alum that lacks the capability to mediate cell-mediated immunity (49), MF59 and AS03 can elicit more balanced immunity, possibly by improving antigen uptake, recruiting immune cells, and promoting the migration of activated antigen-presenting cells (28, 50, 52). Emulsion adjuvants have already been used in preclinical studies of vaccines against coronavirus. MF59 used in inactivated SARS and MERS vaccines, as well as vaccines containing the RBD domain of the MERS-CoV spike (S) protein, has exhibited excellent adjuvanticity, with potent humoral immune responses, *i.e.*, high titers of neutralizing antibodies, and cell-mediated immunity in the coronavirus vaccines (53–55). In addition, depending on the types of antigen, cell-mediated immunity induced by MF59 differs. When formulated with the MERS-CoV S protein, MF59 enhanced both effective CD4^+^ and CD8^+^ T-cell immune responses. In comparison, when combined with inactivated SARS CoV, MF59 induced significant CD4^+^ T cell, but not CD8^+^ T cell responses (56, 57). However, in another study by Zhang et al., it was demonstrated that when MERS S protein was adjuvanted with MF59, it induced higher IgG_1_ and IgG_2a_ antibodies with a slightly Th2-biased response (54). Subsequent studies also showed that ferritin-based MERS-CoV S protein, adjuvanted with MF59, promoted multiple antibody responses, including high levels of IgA antibody titers that resulted in potent mucosal immune responses (58). A study by Tang *et al.* has indicated that there are no significant differences in the neutralizing activity of the serum derived from mice immunized with MERS S377-588 at 1, 5, and 20 μg in the presence of MF59, suggesting the dose-sparing effect of MF59 when it was formulated with MERS S protein (57). However, an immunopathologic lung reaction, as well as an increase in IL-5 and IL-13 cytokines, was seen in animal studies using both MF59-adjuvanted and adjuvant-free inactivated MERS-CoV vaccines (53). It has shown that eosinophil infiltrations with higher Th2-type cytokine secretion aggravated the hypersensitivity-type pulmonary immunopathology when vaccinated with MF59-adjuvanted inactivated virus vaccines as compared with the inactivated virus vaccines alone (53).

Another emulsion adjuvant, AS03, elicits both potent humoral and cellular immune responses to an inactivated whole virion SARS-CoV (WI-SARS) vaccines (59) compared with the virion without adjuvants. Moreover, in the presence of the AS03 adjuvant, an identical trend toward specific CD4^+^ T cell responses was observed when immunized with SARS-CoV containing the equivalent of 0.5 or 1.5 μg of S protein (59). Therefore, the addition of AS03 tends to potentiate the immune responses with a lower dosage of antigen. Considering its capability to induce both arms of the immune system, S protein, RBD domain, and N protein can also be formulated with AS03. Currently, GSK is sharing its AS03 adjuvant with COVID-19 vaccine developers globally (29).

Besides MF59 and AS03, other emulsion-based adjuvants such as Freund’s adjuvant and Montanide ISA51 have also been formulated in CoV vaccines (54). By evaluating the titers of specific serum antibody responses, it has been demonstrated that Freund’s adjuvant and ISA51 elicited significant Th1 antibody responses (IgG_2a_) with no clear Th2 responses (IgG_1_) (54, 59).

## TLR Agonists and Other Adjuvants

Toll-like receptors (TLRs), a category of pattern-recognition receptors, are critical to pathogen recognition. This allows for rapid activation of innate immunity, and subsequently, effective adaptive immunity. TLR agonists have been extensively studied as vaccine adjuvants (60, 61). CpG, Poly I:C, glucopyranosyl lipid A (GLA), and resiquimod (R848) are agonists for TLR9, TLR3, TLR4, and TLR7/8, respectively. These adjuvants have been evaluated in candidate vaccines against SARS CoV (62, 63).

In addition to neutralizing antibodies and CD4^+^ T cells, optimal protection against coronavirus probably involves the synergistic effect of CD8^+^ T cells (64). Memory CD8^+^ T cells solve the problem of neutralizing antibodies only existing for short periods and providing long-term protective cellular immunity (64). Among the TLR agonists, CpG significantly augments the CD8^+^ T cell immune response higher than the others (63). Indeed, it has been demonstrated that CpG can also stimulate enhanced IgG production in animals immunized with an inactivated SARS-CoV vaccine (62). In addition to IgG, IgA production was also enhanced, only when CpG was administered *via* intranasal (*i.n.*) administration (62), indicating immune activation in the mucosal compartment. Although CpG is capable of inducing both cellular and humoral immune responses, it preferentially induces responses that are Th1-biased. Moreover, CpG can divert pre-existing Th2 responses to a Th1 phenotype, which has laid a foundation for the combination of CpG with other adjuvants, most commonly alum (65). In SARS-CoV or MERS-CoV subunit vaccines, studies have found that the combination of alum and CpG elicited higher neutralization antibody titers and a more robust cellular immune response compared with alum alone or alum with other TLR agonists (18, 19). In addition to alum, CpG is combined with Montanide ISA-51, a type of water-in-oil emulsion adjuvant. When the combined adjuvants were formulated with SARS S or N protein, they were capable of promoting robust neutralizing antibody production (66). However, vaccinated with only SARS N protein, animals showed immune responses biased dramatically toward Th1 (67). In addition, it is reported that R848 could enhance antigen-specific CTL response and induce a fast, robust and durable IFN-α production *in vivo* among humanized mice, which is distinct from the experimental findings based on common mouse models (68). However, further studies on R848 adjuvanticity should stress more on vaccine formulation. A recent study by Gadd *et al.* indicated that only when R848 was conjugated with DOPE (1,2−di−(9Z−octadecenoyl)−sn−glycero−3−phosphoethanolamine):DDA (dimethyldioctadecylammonium bromide salt) multilamella liposomes rather than linear mixed, a high potency of immunostimulatory activity was observed (69). Moreover, an R848-encapsulating PLGA nanoparticle can bring down the excessive level of inflammatory cytokines induced by free R848, which could be benefit to provide long-term safety and appropriate immune response (70).

Although CpG had been shown to exhibit considerable potential as a coronavirus-specific adjuvant, studies have found that it might be a poor inducer of long-term immune memory (46). A recent study indicated that single-stranded RNAs (ssRNAs) derived from the Cricket paralysis virus (CrPV) intergenic region (IGR) internal ribosome entry sites (IRES) could function as vaccine adjuvants endowing long-lasting immunity. This adjuvant significantly activates innate immune response through activating TLR7 and enhancing the chemotaxis of professional antigen-presenting cells (APC) (71). Moreover, some novel adjuvants such as STING agonist, Advax, and AS01_B_, which is an adjuvant formulated in recombinant zoster vaccine Shingrix, exhibit advantages for long-lasting immune responses (46, 59, 72). Advax, a delta inulin microparticle adjuvant, augmented the induction of neutralizing antibodies along with the existence of memory B cells and a robust, long-lasting T-cell IFN-γ response when it was formulated in recombinant or inactivated SARS-CoV vaccines (46). Moreover, Matrix M1, a saponin-based adjuvant, has been demonstrated to be more effective than alum adjuvant in inducing neutralizing antibodies to SARS S protein or MERS S protein (17). This might address the concern that S protein may lead to antibody-dependent enhancement (ADE), which is more likely to be triggered by mild antibody production (36).

The SARS-CoV-2 infections occur at the mucosal surface of the upper respiratory tract (73). Thus, the elicitation of protective immune responses at the mucosa is critical. TLR agonists, such as flagellin (74) and CpG ODN (62), have been used as mucosal adjuvants. As discussed above, the CpG ODN can elicit neutralizing antibodies in mucosal compartments (62) when formulated with inactivated SARS-CoV. Additionally, the STING agonist, bis-(3′,5′)-cyclic dimeric guanosine monophosphate (c-di-GMP or cdGMP), has been reported as a potent mucosal vaccine adjuvant that induces Th1 and Th17 cytokines in a plant-derived H5 influenza vaccine after intranasal vaccination (75). In a very recent study, it was demonstrated that pulmonary surfactant–biomimetic liposomes encapsulating STING agonists could be used as mucosal adjuvants for universal influenza vaccines that trigger rapid humoral and cellular immune responses and exhibit sustained cross-protection against influenza (76). Though cdGMP in polymeric nanoparticle formulations has been used as adjuvants with MERS-CoV S-RBD protein, its ability to induce mucosal immunity was not specifically examined (72). Thus, further studies are warranted to examine both the efficacy and safety of mucosal adjuvants in coronavirus vaccines.

## Conclusion and Perspectives

In this article, we provided an overview of previously studied adjuvants in candidate inactivated and subunit coronavirus vaccines with a focus on the types of adjuvants in the vaccine formulations and the nature of immune responses to the formulated vaccines. These previous studies provided a convenient basis for the screening of adjuvants required to develop coronavirus vaccines. In-depth reviews of the various adjuvants, a comprehensive understanding of their impacts on the extent and types of immune responses, and an exploration of their combinations with various antigen types and vaccine platforms will facilitate the selection of adjuvants that provide the required immunological protection of coronavirus vaccines.

In the absence of a cure for COVD-19, effective and safe vaccines are urgently required. Adjuvants such as aluminum-based salts, TLR agonists, emulsions, and other novel adjuvants have distinctive physicochemical properties, which can be significant in regulating the strength, duration, and types of immune responses (19, 63, 77). Studies have suggested that neutralizing antibodies are critical for immune protection (34, 42).While mechanistic studies are still being conducted, emerging evidence has suggested that SRAS-CoV-2-specific CD4^+^ and CD8^+^ T cells in coordination with neutralizing antibodies are required for generating protective immunity against SARS-CoV-2 (33). Thus, the appropriate adjuvants should be selected to formulate specific antigens that will achieve optimal immunogenicity profiles. Current available studies have demonstrated the feasibility of formulating S protein, RBD domain, M protein and N protein with specific adjuvants.

It should be noted that the development of a COVID-19 vaccine has been on a fast track. Thus far, four non-replicating viral vector vaccines, three inactivated vaccines and two mRNA vaccines being under clinical phase III stage, with more are on the way (32). Though different types of adjuvants have been used in exploratory and pre-clinical studies (**Tables 1**–**3**), considering the need for rapid deployment of COVID-19 vaccines for the pandemic, alum, which had been formulated in many other licensed vaccines, have been prioritized (15, 16). In addition to the adjuvants described above, engineered nanomaterials also shed light adjuvant development. It has been shown that physicochemical characteristics of aluminum oxyhydroxide could affect the optimal immunogenicity profiles of vaccine formulations (41, 83, 84). Moreover, a recent study has shown that an alum-stabilized Pickering emulsion (PAPE) showed robust RBD-specific IgG_1_ and IgG_2a_ titers and a high level of inducing IFN-γ-secreting T cells in a COVID-19 vaccine. Additionally, it has been shown that a natural and potent STING agonist encapsulated by pulmonary biomimetic liposomes triggered rapid humoral and cellular immune responses and exhibited a sustained cross-protection against influenza (76). However, more comprehensive mechanistic studies, including the nature of protective immune responses and screening of the various combinations of antigens and adjuvants, are needed for the successful development of a safe and effective COVID-19 vaccine.

**Table 2:** Emulsion adjuvants used in the coronavirus vaccine formulations under exploratory and pre-clinical investigations.

Adjuvant Type	Platform	Adjuvant	Antigen	Antigen Dose	Immunological response								Route	Immunization Schedule	Safety	Animal model	Ref.
					Nab	IgG_1_	IgG_2a_	IgA	Th1 cytokines	Th2 cytokines	Th17 Cytokines	CD8^+^ T cell response					
Emulsion adjuvants	Inactivated vaccines	MF59-like	Inactivated MERS virus	100 μl (1×10^7^ TCID_50_/ml)	√	N/A	N/A	N/A	√	√	N/A	N/A	i.m.	2	Lung immunopathology	hCD26/DPP4 Tg mice	(53)
	Inactivated vaccines	MF59	Inactivated SARS virus	5 μg	√	N/A	N/A	N/A	√	N/A	N/A	×	i.m.	2	N/A	BALB/c mice	(56)
	Inactivated vaccines	AS03	Inactivated SARS virus	100 μL of WI-SARS containing the equivalent of 0.5/1.0/1.5 μg of S protein	√	N/A	N/A	N/A	√	N/A	N/A	×	i.m.	2	N/A	BALB/c mice	(59)
	Subunit vaccines	MF59	MERS-CoV S-RBD-Fc protein	10 μg	√	√	√	N/A	×	N/A	N/A	×	s.c.	3	N/A	BALB/c mice; Ad5-hDPP4 mice	(54)
	Subunit vaccines	MF59	MERS-CoV S-RBD-Fc protein	1/5/20 μg (optimal: 1 μg)	√	√	√	N/A	√	N/A	N/A	√	s.c.	3	N/A	BALB/c mice	(57)
	Subunit vaccines	MF59-like	MERS-CoV S-RBD-Fc protein	10 μg	√	N/A	N/A	N/A	N/A	N/A	N/A	N/A	i.m.	2	Weight loss and death of mice	hCD26/DPP4 Tg mice	(78)
	Subunit vaccines	MF59	MERS-CoV S-RBD-Fc protein	10 μg	√	√	√	N/A	N/A	N/A	N/A	N/A	s.c.	2	N/A	BALB/c mice; hDPP4-Tg mice	(79)
	Subunit vaccines	MF59-like	MERS-CoV S-RBD-Fc protein	10 μg	√	N/A	N/A	N/A	√	N/A	N/A	N/A	i.m.	2	N/A	CD26/hDPP4 Tg mice	(55)
	Subunit vaccines	MF59	MERS-CoV S-RBD-Fd protein	10 μg	√	√	√	N/A	N/A	N/A	N/A	N/A	s.c.	4	N/A	BALB/c mice; hDPP4-Tg mice	(80)
	Subunit vaccines	MF59-like	MERS-CoV S-RBD protein NPs	20 μg	N/A	√	√	√	√	N/A	N/A	N/A	i.m.	3	N/A	BALB/c mice	(58)
	Subunit vaccines	MF59-like	MERS-CoV S-RBD protein	10 μg	N/A	√	√	N/A	×	N/A	N/A	×	s.c.	2	N/A	C57BL/6 mice	(72)
	Subunit vaccines	MF59-like	MERS-CoV/SARS-CoV/SARS-CoV-2 RBD-sc-dimer	10 μg	√	N/A	N/A	N/A	×	×	N/A	×	i.m.	3	N/A	BALB/c mice	(23)
	Subunit vaccines	Montanide ISA51	MERS-CoV S-RBD-Fc protein	10 μg	√	×	√	N/A	×	N/A	N/A	×	s.c.	3	N/A	BALB/c mice; Ad5-hDPP4 mice	(54)
	Subunit vaccines	Freund's adjuvant	MERS-CoV S-RBD-Fc protein	10 μg	×	×	√	N/A	×	N/A	N/A	×	s.c.	3	N/A	BALB/c mice; Ad5-hDPP4 mice	(54)
	Subunit vaccines	Ribi adjuvants	MERS-CoV S1 protein	10 μg	√	√	√	N/A	N/A	N/A	N/A	N/A	i.m.	2	N/A	BALB/c mice	(22)
	Subunit vaccines	Alum- stabilized Pickering emulsion (PAPE)	Recombinant RBD of SARS-CoV-2	5 μg	N/A	√	√	N/A	√	√	N/A	N/A	i.m.	2	Acceptable biosafety	BALB/c mice	(81)

Annotation:

**MF59 adjuvant** an emulsion adjuvant composed of an oil phase (squalene:4.3%): and an aqueous phase (polysorbate 80:0.5%, sorbitan trioleate:0.5%);

**MF59-like** (AddaVax): a squalene-based oil-in-water nano-emulsion based on the formulation of MF59 (squalene:5%, polysorbate 80:0.5%, and sorbitan trioleate:0.5%);

**AS03 adjuvant**: an emulsion adjuvant composed of an oil phase (10.69 mg squalene, 11.86 mg DL-α-tocopherol) and an aqueous phase (4.86 mg polysorbate 80) each 0.5-mL adult dose;

**Ribi adjuvant**: an oil-in-water emulsion containing 2% squalene-Tween 80-water, 0.5 mg monophosphoryl lipid A, and 0.5 mg synthetic trehalose dicorynomycolate;

**Montanide ISA-51**: a water-in-oil (w/o) emulsion adjuvant composed of a mineral oil and a surfactant from the mannide monooleate family;

**Freund’s adjuvant**: heat-killed mycobacterium tuberculosis in non-metabolizable oils (paraffin oil and mannide monooleate);

**Nab** represents the neutralizing antibodies, and N/A means not available.

**Table 3:** TLR agonists and other adjuvants used in the coronavirus vaccine formulations under exploratory and pre-clinical investigations.

Adjuvant Type	Platform	Adjuvant	Antigen	Antigen Dose	Immunological response								Route	Immunization Schedule	Safety	Animal model	Ref.
					Nab	IgG_1_	IgG_2a_	IgA	Th1 cytokines	Th2 cytokines	Th17 Cytokines	CD8^+^ T cell response					
TLR agonists	Inactivated vaccines	CpG ODN 2006	inactivated SARS-CoV(SARS-CoV Z-1 strain virus)	10 μg	√	N/A	N/A	N/A	N/A	N/A	N/A	√	i.n./i.p.	3	N/A	BALB/c mice	(62)
	Subunit vaccines	Monophosphoryl lipid A	MERS-CoV S-RBD-Fc protein	10 μg	√	√	×	N/A	×	N/A	N/A	×	s.c.	3	N/A	BALB/c mice; Ad5-hDPP4 mice	(54)
	Subunit vaccines	CpG ODN 1826	SARS S peptide (HLA-A*0201 restricted)	20 μg	N/A	N/A	N/A	×	×	N/A	√	N/A	s.c.	2	N/A	HLA-A*0201 Tg mice	(63)
	Subunit vaccines	Poly(I:C)	SARS S peptide (HLA-A*0201 restricted)	20 μg	N/A	N/A	N/A	×	×	N/A	√	N/A	s.c.	2	N/A		(63)
	Subunit vaccines	R848	SARS S peptide (HLA-A*0201 restricted)	20 μg	N/A	N/A	N/A	×	×	N/A	√	N/A	s.c.	2	N/A		(63)
	Subunit vaccines	CpG ODN 1826 + Alhydrogel	SARS S protein	8 μg	√	√	√	√	√	N/A	N/A	√	s.c.	2	N/A	129S6/SvEv mice	(19)
	Subunit vaccines	CpG+aluminum oxyhydroxide	MERS S protein	10 μg	√	√	√	√	√	N/A	N/A	√	i.m.	3	N/A	BALB/c mice	(18)
	Subunit vaccines	CpG+ Montanide ISA-51	SARS S protein	30 μg	N/A	N/A	N/A	N/A	N/A	N/A	N/A	N/A	s.c.	3	N/A	BALB/c mice	(66)
	Subunit vaccines	CpG+ Montanide ISA-51	SARS N protein	50 μg	N/A	√	√	√	N/A	N/A	√	N/A	s.c.	3	N/A	BALB/c mice; Macaques	(67)
	Subunit vaccines	GLA+ Alhydrogel or aluminum phosphate	RBD (S318-510) of the SARS-CoV S protein	N/A	√	√	√	N/A	N/A	N/A	N/A	√	i.m.	2	N/A	BALB/c mice	(82)
	Subunit vaccines	ssRNA+ aluminum hydroxide	MERS-CoV S protein	1 μg	√	√	N/A	√	N/A	N/A	√	√	i.m.	2	N/A	hDPP4 Tg mice	(71)
Others	Inactivated vaccines	AS01_B_	SARS inactivated whole virus	0.5/1 μg	√	N/A	N/A	N/A	N/A	N/A	×	√	i.m.	2	N/A	BALB/c mice; Hamsters	(59)
	Inactivated vaccines /Subunit vaccines	Advax	SARS S protein/inactivated whole virus	1 μg	√	√	√	√	√	√	√	√	i.m.	2	N/A	BALB/c mice	(46)
	Subunit vaccines	Matrix M1	SARS S protein/MERS S protein	1/3/10 μg	√	N/A	N/A	N/A	N/A	N/A	N/A	√	i.m.	2	N/A	BALB/c mice	(17)
	Subunit vaccines	NP-cdGMP	MERS-CoV S-RBD protein	10 μg	N/A	√	√	N/A	√	√	√	√	s.c.	2	N/A	C57BL/6 mice	(72)

Annotation:

**MPLA** (monophosphoryl lipid A): a low-toxicity derivative of lipopolysaccharide (LPS), that retains the immunologically active lipid A portion of the parent molecule;

**CpG ODN**: synthetic oligodeoxynucleotides containing unmethylated CpG motifs;

**PolyI:C** (Polyinosinic-polycytidylic acid): a synthetic analog of double-stranded RNA (dsRNA), a molecular pattern associated with viral infection;

**R848** (resiquimod): a small molecular weight imidazoquinoline compound, an immune response modifier with potent antiviral and antitumor activities;

**GLA**: glucopyranosyl lipid A, a synthetic Toll-like receptor 4 (TLR4) agonist;

**Advax**: a novel microcrystalline polysaccharide particle engineered from delta inulin;

**AS01_B_**: a liposome-based emulsion adjuvant system containing two immunostimulants, 3-O-desacyl-4′-monophosporyl lipid A (MPL) and the saponin QS-21;

**Matrix M1**: consists of two individually formed 40-nm-sized particles, each with a different and well-characterized saponin fraction (Fraction-A and Fraction-C);

**NP-cdGMP**: cyclic diguanylate monophosphate (cdGMP), a canonical STING (stimulator of interferon genes) agonist, encapsulated into PLGA-based hollow nanoparticles.

**Nab** represents the neutralizing antibodies, and N/A means not available.

## Author Contributions

ZLiang, HZhu, XW, BJ, and XX wrote the manuscript. BS, LS and HZeng conceived and revised the manuscript. HS, YY, and WZ provided critical suggestions. All authors contributed to the article and approved the submitted version.

## Funding

This work was supported by the National Natural Science Foundation of China (31870919), Natural Science Foundation of Liaoning Province (No. 20180550597), LiaoNing Revitalization Talents Program (XLYC1807113), Dalian Science and Technology Innovation Fund (No. 2020JJ25CY015), and Fundamental Research Funds for the Central Universities (DUT19TD12).

## Conflict of Interest

WZ was employed by NCPC Genetech Biotechnology Co., Ltd. LS was employed by Shanghai Zerun Biotechnology Co., Ltd.

The remaining authors declare that the research was conducted in the absence of any commercial or financial relationships that could be construed as a potential conflict of interest.
